# The National Dispensatory

**Published:** 1884-11

**Authors:** 


THE NATIONAL DISPENSATORY, Containing the Natural History, Chemistry, Pharmacy, Actions and Uses of Medicines, Including those recognized in the Pharmacopœias of the United States, Great Britain and Germany, with numerous references to the French Codex. By Alfred Stillé, M.D. LL,D., Professor Emeritus of the Theory and Practice of Medicine and of Clinical Medicine in the University of Pennsylvania, and John W. Maish, Phar. D., Professor of Materia Medica and Botany in the Philadelphia College of Pharmacy. Third Edition, thoroughly revised, with numerous additions, with three hundred and eleven illustrations. Philadelphia: H. C. Lea's Son & Co., 1884.

While we were more than pleased with the first and second editions the third delights us.

To physicians who have an old or even recent copy of the United States Dispensatory, valuable as it is, this book will prove a revelation indeed. The articles on Cinchona, Opium, Alcohol and Water are incomparably the best in our language, and are worth the price of the volume. The arrangement is alphabetical, the index general and therapeutic. It will be an enduring and towering monument of the medicine of to-day, of the industry and patience of the collaborators and of the wisdom of the authors long after their bones are dust.

We thank them in the name of the *whole* profession for their great work, and hope they may long live to keep it, as they have, fully abreast of the times.

Dr. J. S. Todd, Professor of Materia Medica and Therapeutics in the Atlanta Medical College, among other things, said to the class in his first lecture to them upon the opening of the winter session of this year: "Gentlemen, every one of you must have a copy of the New National Dispensatory. It is not only the best book of its kind, but I regard it as the best single book ever issued on a medical subject. You could better afford to be without even an Anatomy than this work. A new edition has just been issued, and, although I have not yet had the pleasure of seeing it, I know from the thorough and conscientious manner that characterized the second edition and the eminent authors, that it will indeed be a treasure. It is a work that every one of you will be compelled to have when you go out to practice, so get it now. It is, besides being materia medica, therapeutics, chemistry and pharmacy, etc., a general history of medicine."

Eighty new pages of therapeutic matter, and sixteen hundred references in the index, have been added to this edition.

